# Investigating the self-study phase of an inverted biochemistry classroom – collaborative dyadic learning makes the difference

**DOI:** 10.1186/s12909-019-1497-y

**Published:** 2019-02-28

**Authors:** Susanne J. Kühl, Achim Schneider, Hans A. Kestler, Matthias Toberer, Michael Kühl, Martin R. Fischer

**Affiliations:** 1 Institute of Biochemistry and Molecular Biology, Ulm University, Albert-Einstein-Allee 11, 89081 Ulm, Germany; 2 Office of the Dean of Studies, Medical Faculty, Ulm University, 89081 Ulm, Germany; 3 Institute of Medical Systems Biology, Ulm University, 89081 Ulm, Germany; 4 Institute for Medical Education, University Hospital, LMU Munich, 80336 Munich, Germany

**Keywords:** Inverted classroom, Flipped classroom, Self-study phase, Medical course of studies, Biochemistry, Collaborative learning

## Abstract

**Background:**

The inverted classroom approach is characterized by a primary self-study phase for students followed by an on-site, face-to-face teaching phase that is used to deepen the prior acquired knowledge. Obviously, this teaching approach relies on the students preparing before the on-site phase, which in turn requires optimized preparatory material as well as defined working instructions. The major aim of this study, therefore, was to investigate the effect of different preparatory materials and working instructions for the self-study phase of an e-learning-based inverted classroom on the knowledge gained by medical students in biochemistry. Furthermore, we analyzed whether collaborative dyadic learning during the self-study phase is more effective than individual learning with respect to knowledge gain.

**Methods:**

The study was performed in a biochemistry seminar for second semester medical students at Ulm University in Germany. This seminar was held using an e-learning-based inverted classroom. A total of 196 students were divided into three homogeneous study groups that differed in terms of the working material and instructions provided for the self-study phase. Knowledge gain was measured by formative tests at the beginning of the on-site phases. Questionnaires were also handed out asking about motivation, interest and learning time in the self-study phases.

**Results:**

Students who were told to prepare in collaborating dyads during the self-study phase performed better in formative tests taken at the beginning of on-site phases than learners who were told to prepare individually. The study material that was provided was of minor importance for the differences in formative testing since almost all students prepared for the on-site phases. With the dyadic learning approach, both students benefited from this collaboration, characterized by a higher motivation and interest in the topic, as well as a longer time spent on task.

**Conclusion:**

Our study provides strong evidence that the study material, but more importantly the instructions provided for the self-study phase, affect students` knowledge gain in an e-learning-based inverted classroom. The instructed collaboratively working group was the most successful.

**Electronic supplementary material:**

The online version of this article (10.1186/s12909-019-1497-y) contains supplementary material, which is available to authorized users.

## Background

The Inverted Classroom (IC) is a state-of-the-art teaching method based largely on a blended learning approach and is characterized by two distinct phases [1, 2]. In the primary self-study phase, students prepare a certain subject matter on their own followed by an on-site phase in which learners apply the acquired knowledge in a group of students under the supervision of a lecturer. The advantage of this method is that the learning material is initially appropriated in the self-study phase, which creates more free time in the following on-site phase. This freed-up time in the on-site phase can be used to apply the prepared learning matter in a more suitable manner. As a result, passive knowledge is transferred into more active knowledge, resulting in a higher level of learning. Moreover, additional competencies can be gained during the on-site phase [3].

Initial studies have rated the IC method as being conducive to learning [4]. The IC method has already been put to successful use in different fields such as medical studies, dentistry, pharmaceutics, nursing as well as other healthcare professions and has been described as a meaningful teaching approach [5–17]. In previous studies, we and others have observed that students were more motivated and satisfied with the IC method than with traditional teaching methods [3, 17–20].

The IC method can only be implemented successfully if the students prepare for the on-site phase in the initial self-study phase. The better the students prepare, the more effective the method becomes. If students do not prepare, subject matters cannot be applied in the on-site phase. Thus, special attention should be paid to the preparatory material for the students and to the instructions for the self-study phase. We suggested implementing the IC approach with an e-learning-based self-study phase since computer-based learning has become a relatively familiar and well-accepted mode of education for students [3]. This is substantiated by other studies that show a high preference amongst students for digital media compared to non-digital learning resources in a pharmacology course [21]. Furthermore, medical students preferred to learn using computer-aided course instructions instead of paper workbooks in a physiology course [22].

For many years, learning in a group has been described as an effective learning method [23–25]. It has been shown that collaborative learning increases the learning success for cognitive and affective learning objectives, and increases the social competence of learners [26, 27]. Moreover, collaborative learning approaches can have a positive effect on the learner’s intrinsic motivation and internal attitude when it comes to learning certain subjects. It has also been reported that learners benefit from learning in smaller groups [28]. Guidelines on how to structure a computer-based, interactive process through learning partners have already been published [29, 30]. But little information is as yet available on the use and efficacy of collaborative, computer-based learning in the self-study phase of an IC. Moreover, the influence of different learning materials and instructions on a student’s learning success in an IC has not yet been investigated in detail.

The main aim of this study was to analyze the influence of the instruction and study material provided for the self-study phase of an e-learning-based inverted classroom on the knowledge gain of medical students in the field of biochemistry. In particular, we investigated whether collaborative learning dyads of students in the self-study phase achieve higher conceptual and/or conditional knowledge in comparison to individual learners.

## Methods

### Course description

The study was performed in a biochemistry seminar entitled “From gene to protein*”*, as part of the so-called Integrated Seminar. This is a compulsory course for all medical students in Germany in the pre-clinical stage of their studies. In Ulm, this particular course is held in the second semester. The course comprises two appointments (on-site phases 1 and 2) of four hours each. Furthermore, students have to prepare for the appointments (self-study phases 1 and 2). All students are assigned to classes and each class consists of about 20 students.

### Participants in the study and classification

During the summer semester 2017 at Ulm University, 196 s semester medical students participated in this study. Not all of the students completed all of the voluntary questionnaires or took part in the knowledge tests of this study. The exact numbers of individual students who answered particular questionnaires are shown in the legend for each figure or table.

Students are generally assigned to classes by the Office of Student Affairs of the Medical Faculty Ulm for this seminar*.* This is a random group allocation without any influence from the lecturers*.* Some students only swap groups so that they can attend other elective subjects.

### Study design and instructions for self-study phases

**Fig. 1:**
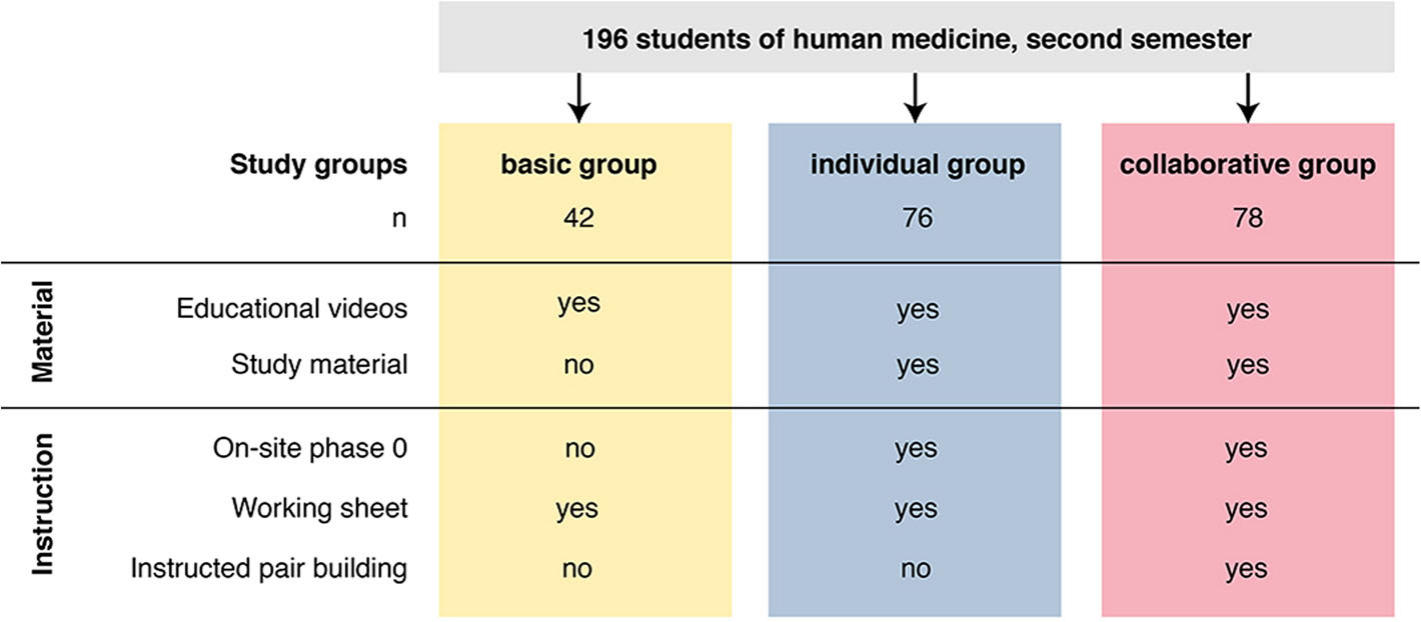
Overview of the independent variables for the different study groups. In summary, 196 medical students from Ulm University participated in this study. The study was carried out in the second semester in a pre-clinical biochemistry seminar. **Yellow:** students in the basic group (*n* = 42) received an information e-mail in which they were told to watch three videos until on-site phase 1 (working sheet basic group). For self-study phase 2, students in the basic group were told to watch two videos. They were neither instructed to form learning dyads, nor did they receive any comprehension questions. **Blue:** 76 students were in the individual group. After an information e-mail, they started with an on-site phase 0. They received instructions and a working sheet (working sheet individual group) for self-study phase 1. Most importantly, they were told to prepare alone in self-study phase 1. Self-study phase 1 was characterized by watching videos and dealing with comprehension questions as provided in the study material. Self-study phase 2 was similar to self-study phase 1. **Red:** 78 students were in the collaborative group. The study procedure for the collaborative group was the same as that of the individual group, except they were told to prepare in learning dyads during the self-study phases

To investigate the influence of the working material and the instructions provided for the self-study phases, we created three random study groups (see Fig. 1). These groups differed in the study material provided for the self-study phases and the accompanying instructions (including the on-site phase 0). Two classes with 42 students in total were assigned at random to the basic group, four classes with 76 students in total to the individual and four classes with 78 students in total to the collaborative dyad group. Questionnaires on the sociodemographic characteristics of participants (sex, age and semester) and prior knowledge (grades of the final secondary-school examinations and the exams in the first semester such as anatomy, biology, terminology, chemistry and physics) were used to check for homogeneous study groups. Furthermore, a questionnaire was used on students´ motivation. All three study groups had access to the same educational videos and instructions to watch these videos before the following on-site phases.

The students in the basic group were briefed by an e-mail to study the three educational videos up to on-site phase 1 (instruction basic group). They received no additional study material with comprehension questions at all.

In an on-site phase 0, both the individual and the collaborative group received additional study material for the self-study phase to support learning. Additionally, they were instructed to learn individually or in collaborative dyads. The students in the individual and collaborative group received worksheets with exact instructions on how to learn during the self-study phases in preparation for either on-site phase 1 or 2. Whereas the students in the individual group were instructed to solve the comprehension questions on their own (instruction individual group), the students in the collaborative group were told to deal with the tasks from the self-study phases in cooperation with a learning partner of their own choice (instruction collaborative group). Learning dyads were created during on-site phase 0 when all of the students were present. Different worksheets were drawn up by S.J.K. and reviewed by two independent experts (M.K. and A.S).

Knowledge tests were taken immediately at the beginning of the on-site phases 1 and 2 to investigate the success of the prior preparation of students.

Additional file 1: Figure S1 and Additional file 2 provide a detailed outline of the study design with respect to the timing of actions (information about the IC method, questionnaires and tests). A total of four teachers were involved in teaching during the on-site phases, whereas one lecturer (S.J.K.) was responsible for providing instruction for all three study groups.

### Material for the self-study phases: educational videos and associated study material

The three educational videos for self-study phase 1 had already been used in our previous study and include the following topics: protein biosynthesis in general (transcription and translation at the ribosome), protein biosynthesis into the rough endoplasmatic reticulum and the subsequent vesicle transport as well as the structure and formation of the collagen triple helix and fibrils [3]. The two educational videos for self-study phase 2 present two biochemical methods: SDS-PAGE (sodium-dodecylsulfate polyacrylamide gelelectrophoresis) and DNA sequencing. All of the educational videos are available from the Institute of Biochemistry and Molecular Biology at Ulm University.

Additional printed study material with eleven (self-vstudy phase 1) and four (self-study phase 2) comprehension questions were provided as a self-study assessment for the individual and collaborative learners. Questions regarding conceptual and conditional knowledge were integrated into the material for each self-study phase. Whereas the comprehension questions in self-study phase 1 only asked for biochemical basics, the study material for self-study phase 2 began with a clinical case report and included one question based on this case. Half of the comprehension questions had already been used in the same seminar the previous year [3]. All of the comprehension questions were drawn up by S.J.K. and reviewed by four independent experts, one internal (M.K, Ulm University) and two external biochemical experts (Universities of Frankfurt and Würzburg, Germany) as well as one psychologist (A.S., Ulm University).

### Ethics and consent

The ethics committee of Ulm University confirmed that no approval was required for this study. Students were verbally informed by a power point presentation during the first on-site phase that all tests and questionnaires were voluntary and anonymous. Students were informed that by handing in the questionnaires, they automatically gave their informed consent. No fees were paid for participation in this study.

### Data collection

Participation in all questionnaires and knowledge tests was voluntary and anonymous. Since one student refused any active involvement in the study, the questionnaires and tests for this person were removed from the study. In addition, because a few statements within the remaining questionnaires were contradictory, a double check was performed on these statements. Statements where no definite attribution was possible were classified as missing, excluded from analyses and therefore led to minor variations in sample sizes (see Figures and tables for the exact number of tests (n)).

#### Determination of knowledge gain

To test the acquisition of knowledge in the self-study phases, formative, written tests were held at the beginning of on-site phase 1 and 2. These tests asked about the content of the self-study phases and had to be answered individually. The tests included questions about conceptual (multiple choice questions) and conditional knowledge (problem-solving questions; [31, 32]). Multiple-choice questions of type A_pos_ (choose one correct answer from five possible answers), A_neg_ (choose one incorrect answer from five possible answers) and K_prim_ (decide for each statement whether it is correct or not) and the competency levels 1 (remembering), 2 (understanding) and 3 (applying) of the Bloom taxonomy were asked for the conceptual knowledge [33, 34]. Problem-solving tasks were asked and students had to write free-text answers for the conditional knowledge. Competency level 4 (evaluate) was reached through these problem-solving questions. Part of the knowledge test 1 had already been used with other students the previous year and had worked well [3]. Both knowledge tests were prepared by a biochemical expert (S.J.K.) and reviewed by four independent experts, one internal (M.K, Ulm University) and two external biochemical experts (Universities of Frankfurt and Würzburg, Germany) as well as one psychologist (A.S., Ulm University). Since the questions for conditional knowledge were answered by the participants with free texts, the responses were rated manually using an expert solution. Rating for all conditional questions was performed by one biochemical expert (S.J.K.) and cross-checked by a second one (M.K.).

### Questionnaires about motivation, interest and learning behavior

In order to analyze the students’ motivation and interest in the material of the self-study phases, questionnaires were issued at the beginning of on-site phases 1 and 2 (second and third data collection). Students were also asked whether they understood the relevance of the material in the self-study phases. All of these questions were rated on a Likert-scale from 1 (strongly disagree) to 6 (strongly agree). The students were also asked about their learning behavior (individual or collaborative learning, learning period, preparation done or not). All questionnaires were drawn up by S.J.K. and reviewed by two independent experts (M.K. and A.S).

### Data analyses and statistics

We conducted an a priori estimation of sample size for all effects we wanted to investigate. A medium effect size of d = 0.6 was assumed for these calculations, power was set to 80% and alpha was set to 5%. The estimated number of total participants was *n* = 111, translating into *n* = 37 for each group respectively. Comparisons of sociodemographic data were carried out between groups with Chi-squared tests for sex and with ANOVAs for age, semester and grades. Analyses of the preparatory material and interest in the self-study phases of the inverted classroom were carried out with ANOVAs between groups, whereas Tukey-HSD was used for post hoc single comparisons. Analyses of the manner of preparation and time taken for the self-study phases of the inverted classroom were carried out with Kruskal-Wallis H tests, whereas Mann-Whitney U tests were used for post hoc single comparisons. The Mann-Whitney U test was also used to determine statistical differences in the knowledge tests.

An assessment of pairs versus individual members was performed using a bootstrap procedure (*n* = 10^6^) on the pairing of all individual participants (students of the individual group) and taking the performance of the more confident member as the pair’s performance. Mean performance values were calculated and compared to the mean values of collaborative group [35].

A *p*-value of < 0.05 was considered to be significant. Statistical significances are as indicated in the legends of all figures.

## Results

### Study groups were homogeneous

To analyze whether study groups were homogeneous with respect to their participants, socio-demographic data (sex, age, semester) and prior knowledge (grades in the final secondary-school examination and 1st semester) was collected for all study groups (Table 1). The data shows that the study groups did not differ significantly with respect to these factors. We also asked about the students` basic motivation and interest (Table 2). This data also indicated a motivation and interest similar to that for studying human medicine or learning biochemistry in the three groups, basic, individual and collaborative.

**Table 1:** Comparison between study groups

	Basic group	Individual group	Collaborative group	Total	Group comparison
	(SD)	(SD)	(SD)	(SD)	
n	42	76	72–75	190–193	
sex (female in %)	71.4	67.5	58.7	64.9	n. s.
					(chi^2^(2, *N* = 193) = 2.30, *p* = 0.32)
age	21.6	21.35	21.76	21.56	n. s.
	(3.37)	(3.28)	(3.99)	(3.57)	(F(2,190) = 0.25, *p* = 0.78)
semester	2.02	1.99	2.04	2.02	n. s.
	(0.35)	(0.11)	(0.26)	(0.24)	(F(2,190) = 0.97, *p* = 0.38)
grade final secondary-school examination	1.48	1.51	1.58	1.53	n. s.
	(0.43)	(0.47)	(0.50)	(0.47)	(F(2,187) = 0.63, *p* = 0.53)
grade exams 1st semester	2.38	2.31	2.30	2.32	n. s.
	(0.80)	(0.55)	(0.65)	(0.65)	(F(2,187) = 0.23, *p* = 0.79)

*n*, number of individual students, *SD* standard deviation, *n.s*. not significant. Between group comparisons were carried out with chi-squared test for sex and with ANOVAs for the other factors

**Table 2:** Basic motivation and interest across different study groups

Items about basic motivation and interest	Basic group	Individual group	Collaborative group	Total	Group comparison
	(SD)	(SD)	(SD)	(SD)	
n	42	76	75	193	
It is for me of great personal importance to study human medicine	5.36	5.51	5.40	5.43	n. s.
	(0.96)	(0.77)	(0.97)	(0.89)	(F(2,190) = 0.46, *p* = 0.63)
I prefer to talk about the content of my study subject than about others subjects	3.33	3.38	3.21	3.30	n. s.
	(0.85)	(1.20)	(1.07)	(1.08)	(F(2,190) = 0.45, *p* = 0.64)
I deal intensively with certain questions of my studies, also independent of examination requirements	3.79	4.01	3.67	3.83	n. s.
	(1.03)	(0.98)	(1.30)	(1.13)	(F(2,190) = 1.85, *p* = 0.16)
I chose my current study mainly because of the interesting topics	5.00	5.10	4.73	4.94	n. s.
	(1.04)	(1.01)	(1.28)	(1.13)	(F(2,190) = 2.14, *p* = 0.12)
My interest in biochemistry is very high	3.57	3.57	3.45	3.53	n. s.
	(1.09)	(1.22)	(1.23)	(1.19)	(F(2,190) = 0.22, *p* = 0.80)
My motivation to learn biochemistry is very high	3.50	3.56	3.33	3.46	n. s.
	(1.04)	(1.23)	(1.30)	(1.22)	(F(2,190) = 0.68, *p* = 0.51)

The items were rated by the students on a Likert-type scale from 1 (strongly disagree) to 6 (strongly agree). *SD* standard deviation, *n.s.* not significant. Between group comparisons were carried out with ANOVAs

We therefore considered the different groups of this study to be homogeneous with regard to the tested factors.

### Students followed the instructions provided

In order to examine the students’ learning behavior in the self-study phases, we monitored how many students prepared for the on-site phases. In the basic group, 87.8% (*n* = 36 answers) watched the educational videos for self-study phase 1 and 100% (*n* = 42 answers) for self-study phase 2. In the individual group, 100% (self-study phase 1; *n* = 76 answers) and 98.6% (self-study phase 2, *n* = 73 answers) prepared for the on-site phases. In the collaborative group, 96% (self-study phase 1; *n* = 72 answers) and 100% (self-study phase 2; n = 72 answers) prepared for the on-site phases. In order to investigate the ecological validity, we verified compliance with the different instructions for the individual and collaborative group. We asked both groups how many students had learned collaboratively with a learning partner. In the individual group, 6.5% (self-study phase 1) and 9.5% (self-study phase 2) had learned with a partner. In the collaborative group, 64% (self-study phase 1) and 58.7% (self-study phase 2) had prepared in a learning dyad as instructed.

This data indicates that nearly all students prepared prior to the on-site phase during the self-study phases and the majority followed the instructions as regards the learning behavior (individual vs. collaborative).

### Collaborative dyadic learning in the self-study phase leads to a better learning outcome

In order to determine whether there was a difference in the learning outcome between the basic, individual and collaborative groups, a knowledge test was carried out at the beginning of on-site phases 1 and 2 respectively. Questions about conceptual (multiple choice questions) and conditional (free text, problem-solving questions) knowledge were integrated in both tests. These tests asked about the content of the self-study phases and had to be completed individually.

**Fig. 2:**
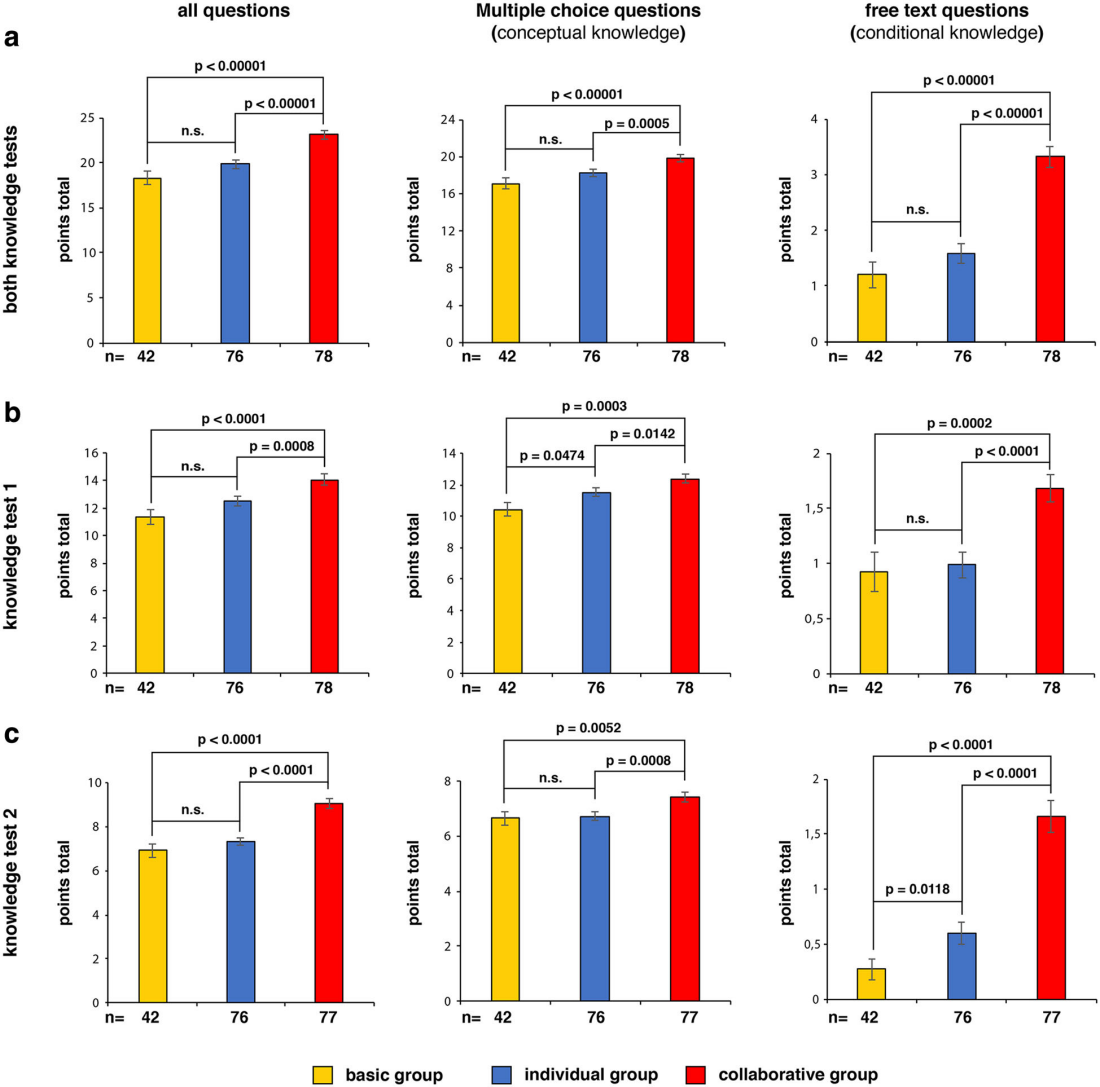
Results of the knowledge tests. **Left:** mean value of the results for all questions. **Middle:** mean value of the results for those questions asked about conceptual knowledge by means of multiple choice questions. **Right:** mean value of the results for those questions asked about conditional knowledge by means of free text questions. The Y-axes show the number of points for each question. **a.** Results of the knowledge test held with the basic (yellow), individual (blue) and collaborative (red) groups after both self-study phases together. **b.** Results of the knowledge test held with the basic (yellow), individual (blue) and collaborative (red) groups after self-study phase 1 (SSP1). **c.** Results of the knowledge test held with the basic (yellow), individual (blue) and collaborative (red) groups after self-study phase 2. n, number of individual students. Standard error of the means are given. The nonparametric Mann-Whitney U test was used for statistical calculations. n.s., not significant

If we consider the results of both tests together (Fig. 2a), students in the collaborative group experienced a significantly higher acquisition of knowledge, divided equally between both conceptual and conditional knowledge, compared to students in the basic and individual groups (Fig. 2a; both *p* < 0.00001). The acquisition of knowledge did not differ significantly between the students in the basic and individual group, nor between conceptual and conditional knowledge. The highest knowledge difference was observed with respect to conditional knowledge between the basic and the collaborative as well as the individual and the collaborative group (Fig. 2a; both *p* < 0.00001).

An analysis of the single knowledge tests revealed the same differences (Fig. 2b and c). In addition, students from the individual group also experienced a significant better acquisition of knowledge compared to the students in the basic group: in self-study phase 1 this was conceptual (Fig. 2b; *p* = 0.0474) and in self-study phase 2 conditional knowledge (Fig. 2c; *p* = 0.0118).

Given the fact that almost all participants prepared for the on-site phases, this data implies that instructions regarding the learning behavior of students (here: individual vs. collaborative) have a greater influence on the learning outcome than the learning material that is provided for the self-study phase.

### Collaborative dyadic learning is more than teaming up with a knowledgeable partner

**Fig. 3:**
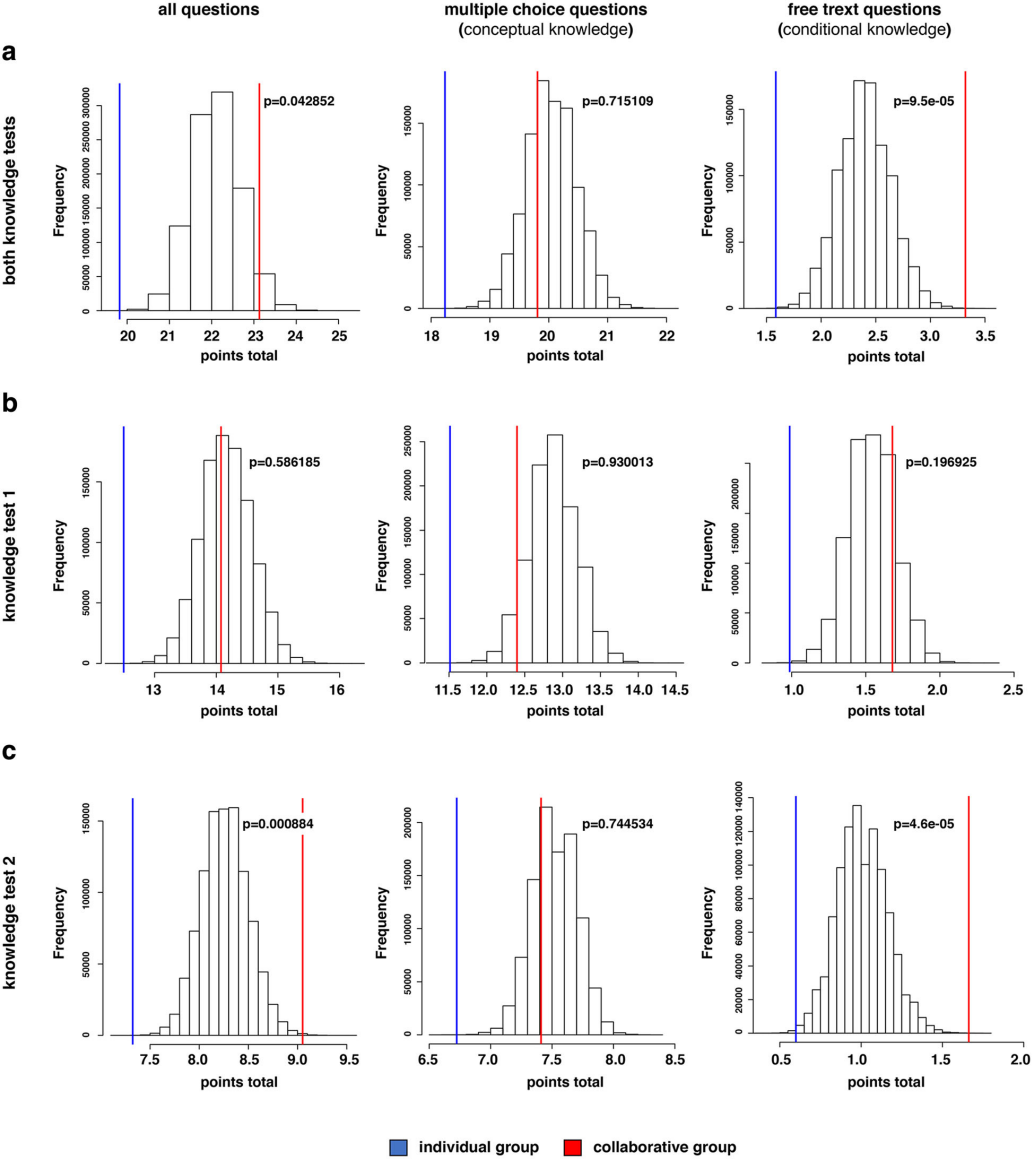
Histograms of the simulated dyads. The distributions of the mean performance values from the bootstrap resampling (*n* = 10^6^) procedure (columns) are shown together with the mean performance of the individual (blue lines) and collaborative groups (red lines). *P* values show the probability of exceeding the mean value of the collaborative dyadic group (one-sided test). **a** Results of both self-study phases. **b** Results of self-study phase 1. **c** Results of self-study phase 2

One possible explanation for the observed effect of superior collaborative preparation could be that dyads of students in the collaborative learning approach perform better because one of the students is more knowledgeable than the other, thereby affecting the learning outcome of the less knowledgeable student. In this scenario, the effect would be due to improved learning of the less knowledgeable member. To test this hypothesis, we generated all possible dyads of participants of students of the individual group (76*75/2 = 2850 possible dyads) and took the performance of the more knowledgeable member in the tests to be the performance of this pair. We then performed an analysis by generating artificial groups of equal size and bootstrap estimated the mean performance of the hypothetical groups generated in this way [36]. The procedure was repeated one million times and the distribution of outcomes was plotted and compared to the means of points achieved in the tests of the individual and collaborative groups (Fig. 3).

This data indicates that the overall learning outcome of the participants in the collaborative group cannot be explained solely by the aforementioned hypothesis. If one considers all of the questions in both tests, the probability of a random group of students performing as suggested above and achieving the same or higher total points in the knowledge tests is only 4% (Fig. 3a; *p* = 0.042852). If one analyzes the questions in more detail, it becomes clear that this effect is only due to questions that test conditional knowledge (Fig. 3 A, B, C, right panels). The probability that the outcome of the test can be explained by the aforementioned hypothesis in this case is less than 0.0095% for both tests (Fig. 3a, right panel; *p* = 9.5e-05).

This data indicates that the observed effect on learning outcomes, in particular with respect to conditional knowledge, entails more than teaming up a more knowledgeable with a less well-trained student, and that both students benefit from collaborative learning. This leads to the assumption that additional effects, such as increased motivation and interest or time on task, contribute to a better outcome of the test, something that we therefore tested next.

### Collaborative dyadic learning stimulates students` motivation and interest

**Fig. 4:**
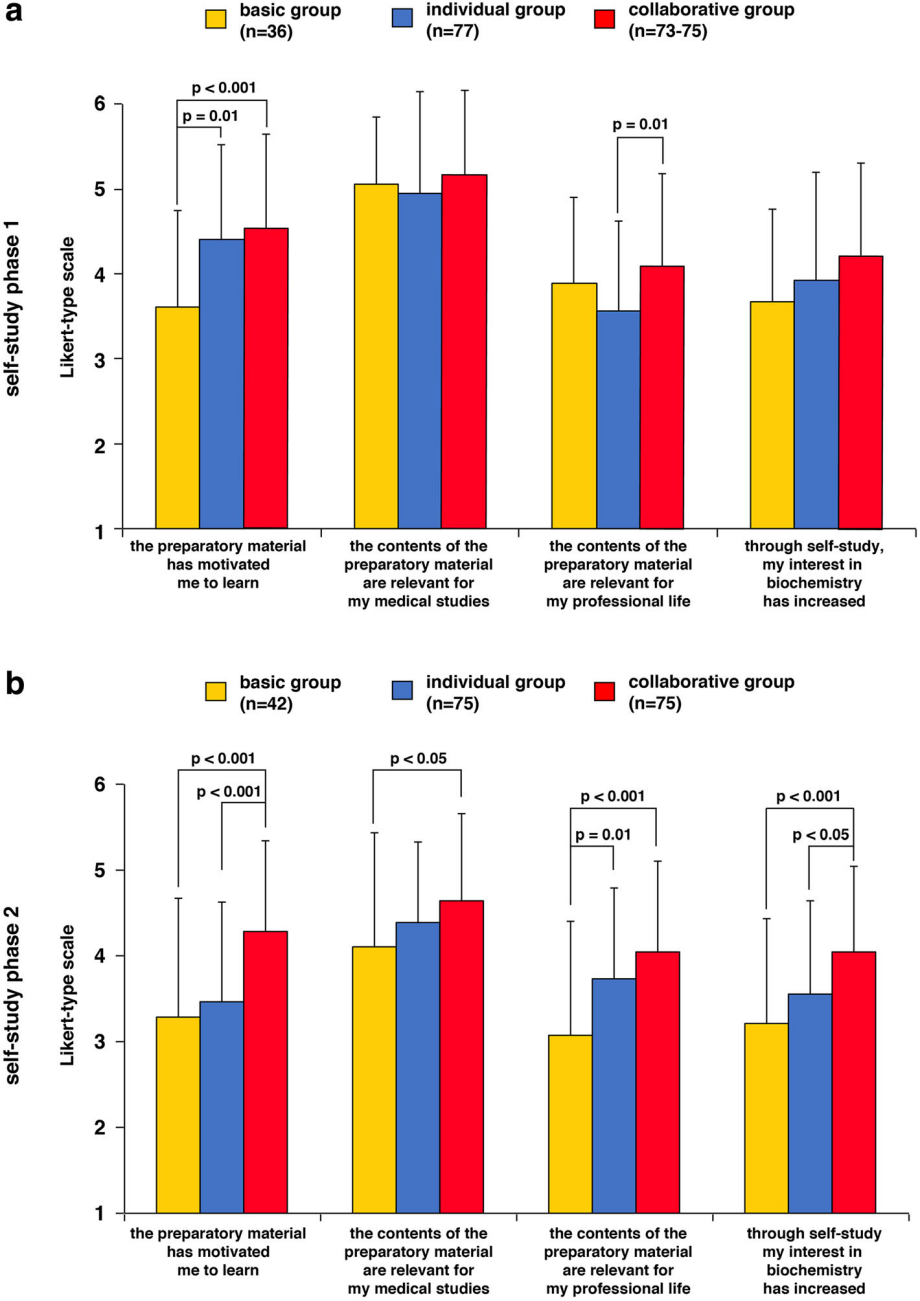
Questionnaires about the preparatory material and interest in the self-study phases. The items were rated by the students on the basis of a Likert-scale from 1 (strongly disagree) to 6 (strongly agree). **a.** Results of self-study phase 1. **b.** Results of self-study phase 2. n, number of individual students. Standard deviations are given. Analyses were carried out with ANOVAs between groups, Tukey-HSD was used for post hoc comparisons

In order to analyze the students` motivation and their interest in biochemistry during the self-study phases, we asked about I) the students` learning motivation, II) their recognition of its relevance for their medical studies or their professional life, and III) their interest in biochemistry (Fig. 4).

In self-study phase 1, the participants in individual and collaborative groups exhibited a significantly higher motivation to learn compared to the basic group (all *p* < 0.01) (Fig. 4a). With respect to their recognition of its relevance for their professional life, the collaborative group scored higher than the individual group (*p* = 0.008).

In self-study phase 2, the collaborative group scored higher than the basic group in every respect (all *p* < 0.05), were more motivated to learn than the individual group (*p* < 0.001) and received a higher score in terms of an increased interest through the self-study phase 2 (*p* = 0.016). The individual group achieved higher values than the basic group with respect to their recognition of its relevance for their professional life (*p* = 0.008) (Fig. 4b).

Taken together, our data indicates that collaborative dyadic learning in the self-study phase increases motivation and an interest in the topic.

### Collaborative dyadic learning affects the time on task

**Fig. 5:**
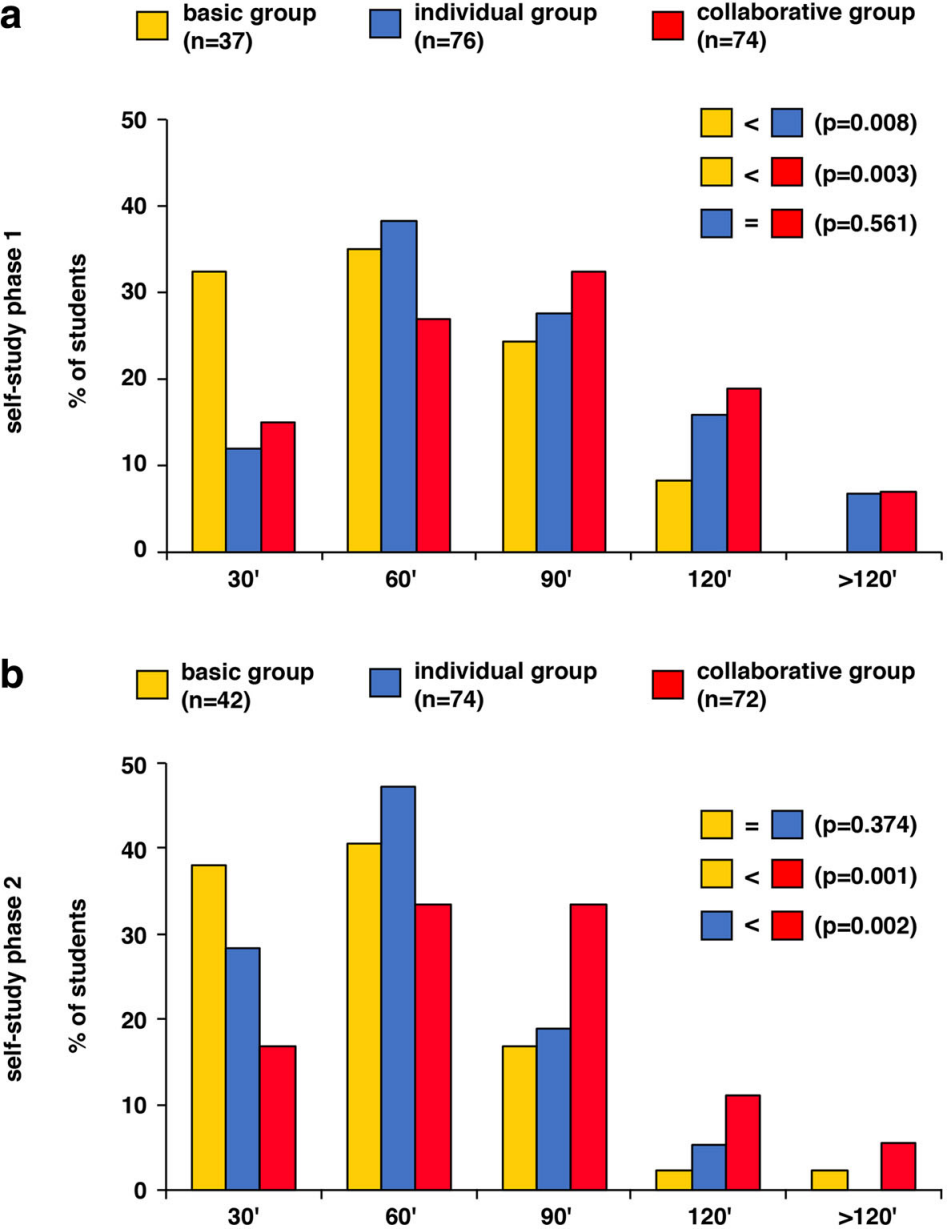
Questionnaires about time on task during the self-study phases. Results of the preparation time in self-study phase 1 and 2 for the students in the basic, individual and collaborative groups. **a** In self-study phase 1, more students in the individual group learned longer than the students in the basic group (*z* (*U*) = 2.65, *p* = 0.008). More students in the collaborative group learned longer compared to the students in the basic group (*z* (*U*) = 2.96, *p* = 0.003). The preparation time of the students in the individual and the collaborative groups did not differ (*z* (*U*) = 0.58, *p* = 0.561). **b** In self-study phase 2, the preparation time of the students in the basic and individual groups did not differ (*z* (*U*) = 0.89, *p* = 0.374). In contrast, more students in the collaborative group learned longer compared to the students in the basic (*z* (*U*) = 3.30, *p* = 0.001) and the individual groups (*z* (*U*) = 3.17, *p* = 0.002). n, number of individual students. Analyses were carried out with Kruskal-Wallis H tests, Mann-Whitney *U* Tests were used for post hoc single comparisons

We also asked about the preparation time in the self-study phases to check for possible differences in learning outcomes between the different study groups (Fig. 5). In self-study phase 1, students in the basic group learned for a shorter period of time than students in the individual and collaborative groups (*p* = 0.008 and *p* = 0.003). In contrast, the learning time in the individual and collaborative groups did not differ significantly (*p* = 0.561). In self-study phase 2, the learning time in the basic and individual groups did not differ (*p* = 0.374). However, students in the collaborative group prepared for longer than students in the basic (*p* = 0.001) and individual (*p* = 0.002) groups.

In summary, students in the collaborative group spent more time on the task than the other groups.

## Discussion

The data obtained from our study for the first time revealed that there is a significant difference in the learning success within an e-learning-based IC approach depending on the learning instructions. Students who were instructed to learn in a dyad (collaborative) were more successful than students who learned individually. In contrast, we discovered a somewhat minor difference in the learning success depending on the learning material provided, as shown by the comparison between the basic and individual groups.

### Learning material for the self-study phase in an inverted classroom setting

We tested the effect of different learning materials on the learning success of students in the self-study phase of the IC. Whereas the basic group were only told to study educational videos, the individual group (as well as the collaborative group) received additional study material including comprehension questions. There was only minor difference between preparation with comprehension questions (individual group) and preparation without comprehension questions (basic group) with respect to the acquisition of both conceptual and conditional knowledge, measured by knowledge tests at the beginning of the on-site phases 1 and 2. A positive effect of the study material on the learning outcome might be obscured in our study. However, since almost all of the students prepared for the on-site phases 1 and 2, this might already indicate a great interest of students in this learning approach. Moreover, the videos may have presented the subject matter in a manner such that the additional study material did not add any significant information. In cases where this cannot be guaranteed, the study material might have a greater impact.

### Learning instruction: collaborative learning as a powerful tool in the self-study phase of an e-learning-based inverted classroom

We were able to show that the learning process in the self-study phase in an IC is dependent upon the instruction given on how to organize the self-study phases. Students who were told to learn with a learning partner were more successful in remembering and applying the content of the self-study phases in the knowledge tests after the self-study phases than students who learned individually. The same effect could also be observed with respect to the conceptual and conditional knowledge. Since almost all of the participants (basic, individual, collaborative group) prepared for the on-site phases 1 and 2, this data implies that instructions regarding the learning behavior of students (here: individual vs. collaborative) have a greater influence on the learning outcome than simply providing additional learning material for the self-study phase.

In short, collaborative preparation in the self-study phase is much more effective than individual preparation. This is not all that surprising as it has already been shown that collaborative learning, such as asking each other questions or explain things to each other (students had to perform both of these tasks in our self-study phases) is presumed to encourage learning [37]. From a socio-cognitive point of view, learning is a change in cognitive structures that consists of new connections being made between new information and prior knowledge, whereby the new information is integrated into the participants’ existing knowledge [38]. According to Vygotsky (1978), this process is greatly affected by interaction and the way activity takes place.

Although research has already shown that several types of collaborative learning activities foster learning, these activities rarely occur in collaborative processes without any structuring of the interaction [37, 39, 40]. Different terms are used in educational literature for structuring interactions such as prompting thinking, scaffolding learning or guiding cognitive performance, to name but a few.

One term that is used more often in combination with computer-supported collaborative learning (CSCL) is scripted collaboration. The term script goes back to Schank and Abelson (1977), who viewed the term script as an internal memory structure of a “sequence of actions that define a well-known situation” (p. 41) [41]. Whereas Schank and Abelson (1977) viewed the term script as being relatively static, the educational view of the term script (or *scripting*) describes it as being externally imposed, more flexible and with a broader application. Scripting collaboration describes externally structured collaborative learning that prompts group interaction, which in turn fosters learning [42–44]. To begin with, the script is imposed on the individual and is therefore considered to be external. Over time and with practice, it will become internalized, following Vygotsky (1978), and therefore can be called an internal collaboration script.

Fischer and colleagues outlined a script theory of guidance for CSCL, which is based on four script components (play, scene, role and scriptlet). These differ with respect to their cognitive target level and seven principles [30]. Their script theory of guidance describes the use of internal scripts, the configuration and reconfiguration of internal scripts and the transfer from external to internal scripts within the seven principles. The theory also describes the advantages of external scripts and states when they are most effective for knowledge acquisition.

In our study, we used script components (play and scene) according to Fischer et al. (2013) to guide the students in the individual and collaborative groups through the self-study phases [30]. It would be interesting to discover whether the learning success of students could be further improved by forming small learning groups and using all four script components in the self-study phases of an IC in future studies. This could be achieved, for example, by setting up closed, virtual interaction rooms and assigning different roles and functions to the participating students. Consequently, collaborative learning in the self-study phase of an IC approach would be taken to the next level, thus allowing more complex interactions between participants.

### Possible reasons for the efficiency of collaborative dyadic learning on learning success

In order to investigate the reason for the better learning success with collaborative dyadic learning, we formed all possible pairs of students of the individual group and used the outcome of the more knowledgeable member as the outcome of this pair. This data revealed that the positive effect of collaborative learning is not due solely to one of the students being more knowledgeable than the other, thus affecting the learning outcome of the less knowledgeable student. Rather, our data shows that both students benefit from one another. This data is in line with other studies, which showed that medical students working in pairs have a better diagnostic performance and this also helps to reveal gaps in knowledge [45, 46]. Dyadic working was also shown to be very efficient for solving mathematical problems, in software development (e.G. *agile* software development and extreme programming) or when learning a foreign language in particular [47–50].

Furthermore, we observed an increase in motivation, interest and recognition of relevance as well as in the length of time on task amongst dyadic learners. This data indicates that the study material, including the comprehension questions, together with the instruction to learn collaboratively, may have a positive effect on motivation and interest. In some respects, students in the individual group showed much more motivation compared to students of the basic group. This demonstrates that the on-site phase 0 may already induce a greater motivation amongst students to prepare for the on-site phase, though this needs to be investigated in more detail in a follow-up study. Moreover, the instruction to prepare collaboratively results in an increase in the number of students who learned in dyads. This effect could be maintained in the second self-study phase, indicating that either the students appreciated this kind of learning or that medical students in the second semester followed the lecturer’s instructions.

### Combination of the inverted classroom with collaborative learning for competency-based training

In a previously published study, we already revealed the benefits of the inverted classroom method for competency-based training [3]. We demonstrated that the freed-up time in the on-site phase can be used to teach competency-orientated learning objectives such as communication in a team or with peers or laymen (medical communication). This was extended in the present investigations to the self-study phase because the collaborative learning approach encourages the mediation of competency-based learning objectives in the following ways. First, the students were actively instructed to form a learning dyad in a self-directed manner, this fostering independent, self-organized team working. Second, the students had to explain the different learning contents to each other. The listeners were allowed to ask for more details, this being tantamount to a simulation of an explanatory meeting with a patient. Third, the students had to cope with problem-solving tasks, a key competency in the correct diagnosis of diseases. Interestingly enough, the positive effect of the collaborative learning method became particularly clear in the problem-solving tasks of the knowledge tests (asking for conditional knowledge). We saw a very significant difference in learning success between collaborative learners and individual learners. Our results support the findings of Hautz and colleagues, who already highlighted the success of pair work in the performance of diagnoses by medical students [45].

### Problem-solving tasks: a powerful instrument to test competency-based learning objectives?

As explained above, the efficacy of dyadic learning could be especially observed in the problem-solving tasks of the knowledge tests (conditional knowledge). Testing competency-based learning objectives calls for appropriate exams and the integration of problem-solving tasks could be one possible solution. The problem, however, is an objective evaluation of student’s free text answers. Since most universities have to deal with quite a large number of medical students each year, the restricted number of teachers will not be able to solve this problem. One possibility could be the implementation of a computer-based/electronic evaluation of free text answers. This is, however, problematic in the case of handwritten answers, which would require character recognition methods for handwritten symbols. Moreover, semantic information extraction systems would have to be developed, e.g. by using deep learning approaches, though these require large corpora of training data [51]. It would be more realistic to develop e-based examinations with long menu options that could be modified and further adapted to allow the possibility of providing free text answers.

### Limitations of the study

The main limitation of our study is the fact that only 64% (self-study phase 1) and 58.7% (self-study phase 2) of students actually prepared in a learning dyad as instructed in the collaborative study group. Nevertheless, we decided to analyze and integrate the data for all students in the collaborative group because this reflects how students follow the teachers` instructions and provides teachers with a more realistic picture of what might be expected from this kind of instruction. The strength of our findings is therefore somewhat underestimated, suggesting that the more students learn in dyads, the better their learning outcome will be.

In our present study, we compared three different study groups. An additional study group with an on-site phase 0 and educational videos as study material, but no comprehension questions in the self-study phases, may have been of interest. We did in fact try to organize such a group. Unfortunately, we were unable to include this data in our analysis because only a very limited number of students attended the on-site phase 0.

Further limitations of the study are that it is a mono-institutional study and that two lecturers are also investigators of the study. Therefore, it would be interesting to replicate this study in another institution and another field of medical education with independent lecturers.

## Conclusion

Our study indicates that the study material and, more importantly, the instructions provided for the self-study phase, affect the success of an e-learning-based inverted classroom. Instructed collaborative dyadic learning had the highest success rate in the learning outcome. It would be interesting to investigate whether the learning success of students could be further improved by forming small learning groups for the self-study phase in the future. Furthermore, a stable electronic evaluation of free text answers should be established to provide a good instrument to test competency-based learning objectives.

## Additional files

Additional file 1: Overview of the study design. (JPG 786 kb)

Additional file 2: Supplementary Material. (DOCX 17 kb)

## Abbreviations

IC: Inverted classroom
n: Number of individual students
SDS-PAGE: Sodium-dodecylsulfate polyacrylamide gelelectrophoresis
