# Supplementary figures and images for: Investigating the self-study phase of an inverted biochemistry classroom – collaborative dyadic learning makes the difference
Source: BMC Med Educ. 2019 Feb 28;19:64. doi: 10.1186/s12909-019-1497-y (PMC6393989; doi:10.1186/s12909-019-1497-y)

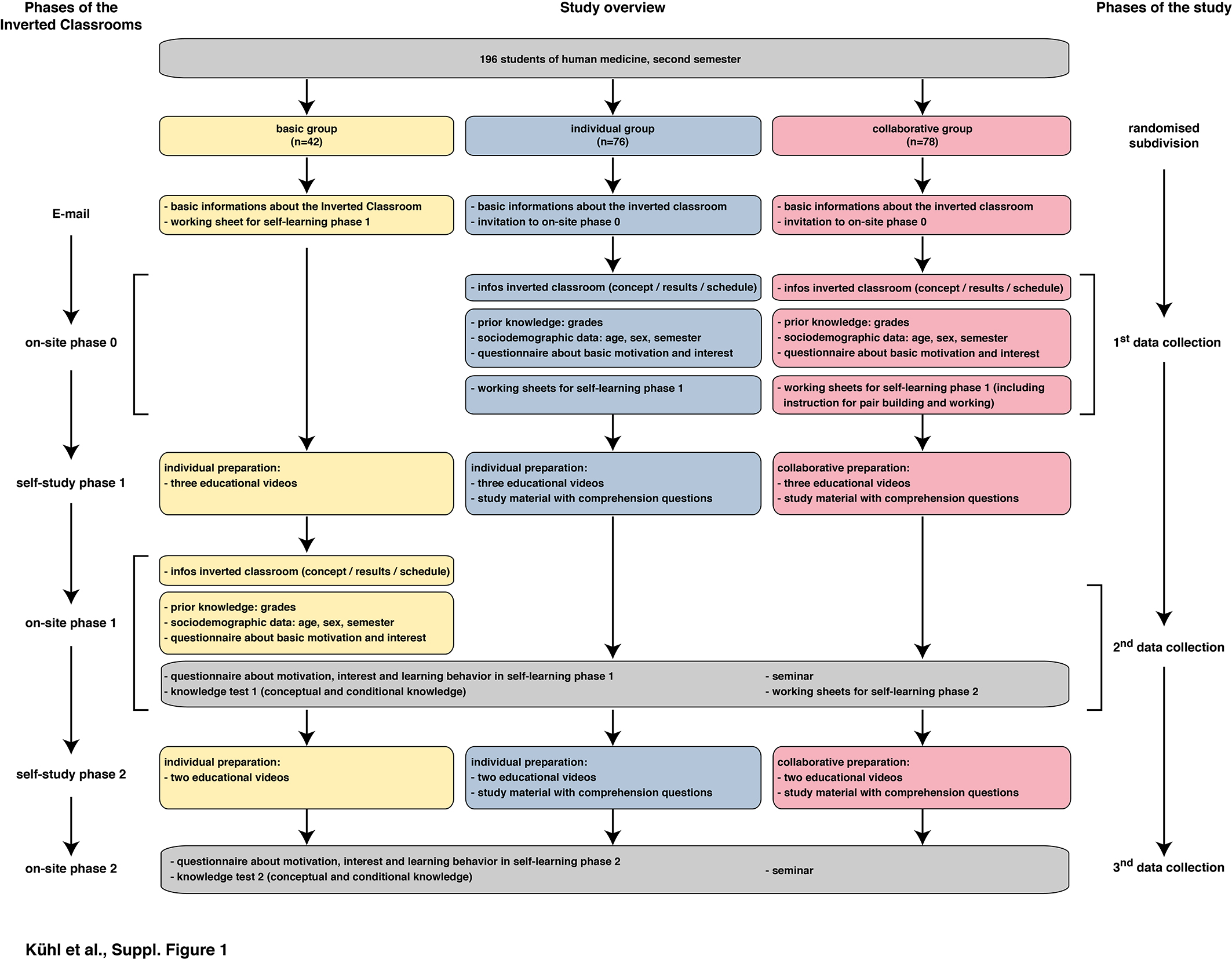

Supplement: Supplementary file 1 — Overview of the study design. (JPG 786 kb) [file 12909_2019_1497_MOESM1_ESM.jpg]
