# Supplementary material for: Investigating the self-study phase of an inverted biochemistry classroom – collaborative dyadic learning makes the difference
Source: BMC Med Educ. 2019 Feb 28;19:64. doi: 10.1186/s12909-019-1497-y (PMC6393989; doi:10.1186/s12909-019-1497-y)
Supplement: Supplementary file 2 — Supplementary Material. (DOCX 17 kb) [file 12909_2019_1497_MOESM2_ESM.docx]

**Supplementary Material**

**Investigating the self-study phase of an inverted classroom – collaborative dyadic learning makes the difference**

Susanne J. Kühl^1,*^, Achim Schneider^1,2^, Hans A. Kestler^3^, Matthias Toberer^1^, Michael Kühl^1^ and Martin R. Fischer^4^

1 Institute of Biochemistry and Molecular Biology, Ulm University, 89081 Ulm

2 Office of the Dean of Studies, Medical Faculty, Ulm University, 89081 Ulm

3 Institute of Medical Systems Biology, Ulm University, 89081 Ulm

4 Institute for Medical Education, University Hospital, LMU Munich, 80336 Munich

* corresponding author

Short title: Analysis of the self-study phase of an Inverted Classroom

**Corresponding author:**

Dr. Susanne J. Kühl

Institute of Biochemistry and Molecular Biology

Ulm University

Albert-Einstein-Allee 11

89081 Ulm, Germany

susanne.kuehl@uni-ulm.de

**Detailed study group and lecturer description**

**Basic group:** Students of the basic group received an e-mail with basic information about the IC method including its advantages. In this e-mail, they were also briefed to study three educational videos until their on-site phase 1 (working sheet basic group). In on-site phase 1, last year’s evaluation results for the seminar as well as the concept of the IC were presented. Furthermore, the schedule for the seminar was explained. Students were informed about the purpose of the study and that participating in the study is voluntary. Afterwards, the students had to fill in questionnaires about prior knowledge (grades), sociodemographic data (age, sex, semester) and basic motivation and interest. Second, a questionnaire about their motivation, interest and learning behavior in self-study phase 1 had to be filled in. As a last action, a knowledge test about the learning content of self-study phase 1 was held (2nd data collection) before the seminar’s subject matter started. At the end of on-site phase 1, students were instructed to study two more educational videos during self-study phase 2 (working sheet basic group). In on-site phase 2, a questionnaire about motivation, interest and learning behavior in self-study phase 2 was handed out and a second knowledge test was held (3nd data collection) before the seminar’s subject matter started.

**Individual group:** Four random classes with a total of 76 students were allocated to the individual group. The course was initiated by an e-mail inviting the students to an on-site phase 0 with a brief explanation (basic information about the IC method including its advantages). A 45-minute introductory event (on-site phase 0) took place 1.5 weeks before on-site phase 1. Last year’s evaluation results for the seminar as well as the IC concept were presented. The schedule for the seminar was then explained. Students were informed about the purpose of the study and that participating in the study is voluntary. Moreover, questionnaires about prior knowledge (grades), sociodemographic data (age, sex, semester) and basic motivation and interest were handed out (1st data collection). At the end, students received working sheets with an exact instruction for self-study phase 1 to study three educational videos and associated comprehension questions. They were instructed to learn in self-study phase 1 on their own in an individual manner (working sheet individual group). On site-phase 1 started with a questionnaire about students´ motivation, interest and learning behavior in self-study phase 1 and a knowledge test 1 about the learning content of self-study phase 1 (2nd data collection) before the seminar’s subject matter started. At the end of on-site phase 1, students received working sheets with an exact instruction for self-study phase 2 to study two educational videos and answer the associated comprehension questions alone (working sheet individual group). In on-site phase 2, a second knowledge test was held at the beginning and a subsequent questionnaire about motivation, interest and learning behavior in self-learning phase was handed out (3nd data collection).

**Collaborative group:** Four random classes with a total of 78 students were assigned to the collaborative dyadic group. Students of the collaborative dyadic group ran through a similar schedule than students of the individual group. The only difference was the instruction for both self-study phases to prepare collaboratively in dyads. The students of the collaborative group received working sheets with an exact instruction for the self-study phases to study the teaching videos and answer the associated comprehension questions together with a learning partner of their own choice (working sheet collaborative group).

**Lecturers**: All introductive E-mails were written by the same lecturer and all on-site phases 0 instructions were given by the same lecturer. The information part and data collection at on-site phase 1 of one basic group was also performed by that lecturer. The information part and data collection at on-site phase 1 of the second basic group was held by a second other experienced lecturer while using the identical presentation slides. On-site phases 1 and 2 including data collection and instruction for self-study phase 2 were held by four equally experienced lecturers.

**Suppl. Figure 1:** Overview of the study design.

In summary, 196 students of the human medicine studies at Ulm University participated at this study. The study was in the second semester in a pre-clinical biochemistry seminar. **Left side:** Different phases of the Inverted Classroom teaching concept. **Middle:** Overview of the study. **Right:** Phases of the study. **Yellow:** 42 students were part of the basic group. These students received an information e-mail. In the following self-study phase 1, they watched three videos. In on-site phase 1, they received detailed informations and filled in questionnaires as well as the knowledge test 1 about the content of self-study phase 1. Self-study phase 2 and on-site phase 2 were similar to self-study phase 1 and on-site phase 1. **Blue:** 76 students were in the individual group. After an information e-mail, they started with an on-site phase 0 in which they received detailed informations and they filled in diverse questionnaires. Also, they were instructed and received a working sheet (working sheet individual group) for self-study phase 1. Most importantly, they were instructed to prepare alone in self-study phase 1. Self-study 1 was characterized by watching videos and dealing with comprehension questions. In on-site phase 1, students had to fill in questionnaires and the knowledge test 1 about the content of self-study phase 1. Self-study phase 2 and on-site phase 2 were similar to the first ones. **Red:** 78 students were in the collaborative dyadic group. Study procedure of the collaborative group was the same than the one of the individual group with the exception that they were instructed to prepare in dyads during the self-study phases.
